# Relapses, Comorbidities, and Predictors of Outcome in Anti-GABA_A_ Receptor Encephalitis

**DOI:** 10.1002/ana.78208

**Published:** 2026-04-12

**Authors:** Claudia Papi, Chiara Milano, Laura Marmolejo, Ana Beatriz Serafim, Esther Aguilar, Mar Guasp, Elianet Fonseca, Mateus Mistieri Simabukuro, Raffaele Iorio, Yuki Fukami, Maria Lucia Schmitz Ferreira Santos, Takashi Miwa, Takashi Araga, Yuto Uchida, Masanori Kurihara, Satoshi Okada, Luz Victoria Garcia, Kenichi Kaida, Natalia Spinola Costa da Cunha, Alberto Vogrig, Florian Lamblin, Juna M. de Vries, Livia Almeida Dutra, Romana Höftberger, Maarten J. Titulaer, Jesús Planagumà, Lidia Sabater, Eugenia Martinez-Hernandez, Thais Armangué, Francesc Graus, Takahiro Iizuka, Josep Dalmau, Marianna Spatola

**Affiliations:** 1 Neuroimmunology Program, Fundacio’ de Recerca Clínic Barcelona-Institut d’Investigacions Biomédiques August Pi i Sunyer (FRCB-IDIBAPS), University of Barcelona and Caixa Research Institute (CRI), Barcelona, Spain; 2 Department of Neuroscience, Catholic University of the Sacred Heart, Rome, Italy; 3 Department of Clinical and Experimental Medicine, University of Pisa, Pisa, Italy; 4 Neurology Service, Hospital Clínic de Barcelona, Barcelona, Spain; 5 Pediatric Neuroimmunology Unit, Neurology Service, Sant Joan de Déu (SJD) Children’s Hospital, ERN-RITA Center, University of Barcelona, Barcelona, Spain; 6 Division of Neurology, Hospital das Clinicas and Câncer do Estado de Sao Paulo, Universidade de Sao Paulo, Sã o Paulo, Brazil; 7 Instituto do Cancer do Estado de Sao Paulo ICESP, Faculdade de Medicina FMUSP, Universidade de Sao Paulo, Sao Paulo, Brazil; 8 Neurology Unit, Fondazione Policlinico Universitario A. Gemelli IRCCS, Rome, Italy; 9 Department of Neurology, Nagoya University Graduate School of Medicine, Nagoya, Japan; 10 Department of Neurology, Hospital Pequeno Príncipe, Curitiba, Brazil; 11 Department of Neurology, National Hospital Organization Osaka Minami Medical Center, Osaka, Japan; 12 St. Marianna University School of Medicine, Kawasaki, Japan; 13 Department of Neurology, University of Tokyo, Tokyo, Japan; 14 Department of Neurology, Ichikawa General Hospital, Tokyo Dental College, Tokyo, Japan; 15 Instituto Nacional de Salud del Niño, Lima, Peru; 16 Department of Neurology, Saitama Medical Center, Saitama Medical University, Kawagoe, Japan; 17 Hospital da Criança de Brasília, Brasília, Brazil; 18 Clinical Neurology, Department of Head-Neck and Neuroscience, Azienda Sanitaria Universitaria Friuli Centrale (ASUFC), Udine, Italy; 19 Department of Medicine (DMED), University of Udine, Udine, Italy; 20 Department of Neurology, University Hospital of La Réunion, Saint-Pierre, France; 21 Department of Neurology, Erasmus University Medical Center, Rotterdam, The Netherlands; 22 Instituto Israelita de Ensino e Pesquisa, Hospital Israelita Albert Einstein, Sã o Paulo, Brazil; 23 Division of Neuropathology and Neurochemistry, Department of Neurology and Comprehensive Center for Clinical Neurosciences and Mental Health, Medical University of Vienna, Vienna, Austria; 24 Centro de Investigación en Red de Enfermedades Raras (CIBERER), Madrid, Spain; 25 Department of Neurology, Kitasato University School of Medicine, Sagamihara, Japan; 26 Department of Neurology, University of Pennsylvania, Philadelphia, PA, USA

## Abstract

**Objectives:**

To characterize the magnetic resonance imaging (MRI) lesion dynamics, comorbidities, predictors of relapse, and outcomes in anti-*γ*-aminobutyric acid type A receptor (GABA_A_R) encephalitis, and assess the utility of LIM-domain-only-protein 5 (LMO5) antibodies as tumor markers.

**Methods:**

GABA_A_R antibodies were confiirmed by 2 techniques in serum or cerebrospinal fluid. Long-term outcomes were defined as good (modified Rankin scale, mRS = 0–1) or poor (mRS 2–5) at ≥12 months. LMO5 antibodies were assessed by cell-based assays and Western blot.

**Results:**

Thirty-three patients were identified (4 children, 29 adults; median age, 5.5 and 60 years; 61% male). Ten patients (10/32, 31%) had concurrent systemic autoimmunity. Adults presented with seizures and cognitive/behavioral symptoms, often with thymoma, gastrointestinal, or other tumors (18/33, 55%), whereas children frequently had seizures and ataxia with cerebellar MRI lesions. Multifocal T2/fluid-attenuated inversion recovery hyperintensities were present at onset in 23 of 31 (74%) or developed later in those with absent or single lesions. Lesions showed dynamic changes, suggesting ongoing inflammation even without clinical correlate. Relapses occurred in 17 of 31 (55%, all adults) and were associated with older age (*p* = 0.02) and lack of second-line immunotherapy (*p* = 0.02). Four patients (4/33, 12%) died. After a 32.5-month median follow-up, 9 of 20 (45%) had persistent cognitive deficits, and 6 of 20 (30%) had a poor outcome, which was associated with relapses (*p* = 0.04). LMO5 antibodies were absent in patients and controls.

**Interpretation:**

Anti-GABA_A_R encephalitis shows age-dependent presentations, most commonly seizures. MRI reveals dynamic changes consistent with an ongoing “clinically silent” inflammation. Relapses and cognitive sequelae are common and associate with not receiving second-line immunotherapy. LMO5 antibodies lack tumor-predictive value.

Anti-*γ*-aminobutyric acid type A receptor (anti-GABA_A_R) encephalitis is a rare autoimmune disorder caused by autoantibodies against the GABA_A_R, a hetero-pentameric ion channel that mediates fast inhibitory synaptic transmission in the central nervous system (CNS).^1–3^ It is characterized by prominent seizures, often refractory to antiseizure medications and evolving into status epilepticus, and multifocal cortico-subcortical cerebral lesions.^1,2,4^ Although acute clinical features have been described in single case reports and few larger cohorts,^1–7^ data on disease evolution over time are limited. Most patients appear to respond initially to immunotherapy, however, relatively short follow-up periods (median, 9 months in the largest series)^1^ make it unclear whether this response is sustained or neurological symptoms persist, resulting in chronic disability. Moreover, although some patients experience symptom recurrence after initial improvement,^6,8,9^ the overall risk of relapse and predictive factors still need to be clarified. MRI abnormalities are also known to change over time,^4^ but their dynamics and clinical implications are poorly characterized.

Tumors, such as thymoma and others,^1,2,10,11^ are known triggers of anti-GABA_A_R encephalitis, occurring in approximately 30% of cases, mainly adults.^1,7^ In anecdotal cases, antibodies against the oncoprotein LIM domain only protein 5 (LMO5)^12,13^ have been found to co-exist in tumor-associated anti-GABA_A_R encephalitis, suggesting they could be used as a biomarker of an underlying tumor.^12,14–17^ However, LMO5 antibodies have not been examined in larger cohorts, and their association with paraneoplastic anti-GABA_A_R encephalitis remains unknown.

In this study, we characterized the clinical and radiological evolution of anti-GABA_A_R encephalitis in a novel cohort of patients, describing MRI dynamics, occurrence of relapses, long-term outcomes, and associated predictors. Additionally, we determined whether LMO5 antibodies could be used to predict a paraneoplastic etiology.

## Methods

### Patients’ Identifcation and Clinical Defnitions

Patients with anti-GABA_A_R encephalitis were retrospectively identified (January 2016–May 2025) from our institutional review board-approved database and biorepository (IDIBAPS R091217, collection C.0006522), which includes clinical information and serum/cerebrospinal fluid (CSF) samples from over 20,000 patients with suspected autoimmune neurological disorders referred to our Neuroimmunology Laboratory (FRCB-IDIBAPS, Barcelona, Spain) for neural antibody studies. Patients were included if they fulfilled the following criteria: (1) clinical picture consistent with encephalitis,^18^ and (2) antibodies to GABA_A_R in their serum and/or CSF detected by rat brain immunohistochemistry and confirmed by a cell-based assay (CBA) with live HEK293 cells expressing α1/ β3 GABA_A_R subunits.^2^ We excluded patients with incomplete clinical data or who had concurrent well-characterized antibodies against other neural intracellular or cell-surface CNS antigens (tested by immunohistochemistry on rat brain or cerebellum, immunodot, and CBA).

Clinical information, provided by referring physicians through a dedicated questionnaire, included demographics, comorbidities, tumor association, prodromal syndrome, neurological symptoms (at onset, during disease course, at relapse, and at last follow-up), paraclinical findings (CSF analysis, electroencephalography [EEG], brain magnetic resonance imaging [MRI], and other ancillary/complementary studies), immunotherapies (including first-line, ie, oral steroids, intravenous methylprednisolone [IVMP], intravenous immunoglobulins [IVIg], plasma exchange [PLEX], and second-line, that is, cyclophosphamide, rituximab, and other immunosuppressants),^19^ oncologic treatments, and outcome at last follow-up (assessed using the modified Rankin scale [mRS]). Brain MRI scans were evaluated as part of routine clinical care by neurora-diologists with expertise in neuroinflammatory disorders at each participating center. Lesion characteristics, including size, anatomical localization, signal abnormalities on T2/fluid-attenuated inversion recovery (FLAIR) sequences, and gadolinium enhancement, were reported by the referring neurologist using a dedicated questionnaire based on the radiology report and review of the MRI images. Tumors were considered as associated with the encephalitis if diagnosed within 2 years (both before and after) from encephalitis onset or at relapse.^20^ Relapse was defined as new onset or worsening of neurological symptoms after a period of stabilization or improvement for at least 2 months.^19^ Long-term outcome and identification of prognostic factors for good (mRS, 0–1) versus poor outcome (mRS, 2–5) were assessed in disease survivors with ≥12 months follow-up from disease onset (and minimum 2 months from last relapse). Predictors of relapse were assessed in all patients alive after the first clinical episode.

### LMO5 Antibody Studies

LMO5 antibodies were assessed with 2 techniques; (1) CBA of HEK293 cells transfected with the human Myc-DDK–tagged LMO5/CSRP2 plasmid (catalog number RC201565, accession number NM_001321, OriGene, Rockville, MD, USA), and (2) Western blot of recombinant LMO5/CSRP2 protein (NBP1-98997, Novus Biologicals, Centennial, CO, USA), as described in Supplementary Material.

### Statistical Analysis

Comparisons between groups (presence vs absence of relapse, good vs poor outcome) were performed using the 2-tailed Fisher exact test (categorical variables) or Mann– Whitney *U* test (continuous variables). Paired observations from the same patient (features at initial episode vs first relapse) were compared using the McNemar test (categorical variables). To identify independent predictors of clinical relapses, statistically significant variables in the univariable analysis were used to build a binary logistic regression model with a stepwise method (forward selection Wald). The IBM SPSS Statistics software and GraphPad Prism v8.0 were used for statistical analyses. Results with *p* < 0.05 were considered statistically significant.

### Standard Protocol Approvals, Registrations, and Patient Consents

The study was approved by the Ethics Committee of FRCB-IDIBAPS (HCB/2023/1169). Written informed consent for the use of samples and clinical information was obtained before participation.

## Results

### Demographics, Tumor Association, and Autoimmune Comorbidities

We identified 33 patients with anti-GABA_A_R encephalitis, including 4 (12%) children (median age, 5.5 years; range, 4–12) and 29 adults (median age, 60 years; range, 23–82), and 20 of 33 (61%) were males.

Individual patient’s demographics, comorbidities, main clinical features, MRI findings, complementary examinations, GABA_A_R antibody status at onset and at relapse, and outcomes at last follow-up are reported in Table S1. GABA_A_R antibodies were detected in both serum and CSF in 25 of 29 patients (87%) with available paired samples, whereas 3 (10%) were positive only in serum, and 1 (3%) only in CSF. The remaining 4 patients had only serum (2) or CSF (2) available, all positive for GABA_A_R antibodies (Table S1).

Nineteen tumors were identified in 18 of 33 (55%) patients, all adults; 1 patient had 2 tumors. Thymoma was the most frequent (13/19, 68%), followed by gastro-intestinal tumors (3/19, 16%; including 1 colorectal cancer, 1 gastric gastrointestinal stromal tumors, 1 esophageal cancer), prostate adenocarcinoma (2/19, 11%), and multiple myeloma (1/19, 5%). Tumors were diagnosed within 2 years of encephalitis onset (18/19) or at relapse (1/19, 3 years after onset): 5 (26%) preceded and 14 (74%) coincided with or followed neurological symptom onset. Three tumors had been previously treated when encephalitis developed. Notably, 1 patient with multiple myeloma had undergone an autologous stem cell transplant 1 year earlier. Among 16 patients with available information, 15 (94%) received oncologic therapy, whereas 1 (1 thymoma) did not.

Of 32 patients with available comorbidity data, 10 (31%, including 6 with associated tumors) had concomitant autoimmune diseases: chronic thyroiditis (n = 5, 2 patients with an additional diagnosis of multiple sclerosis and pernicious anemia, respectively), myasthenia gravis with AChR antibodies (n = 2), pemphigus (n = 1), systemic lupus erythematosus (n = 1), Crohn’s disease (n = 1). The patient with multiple sclerosis had received alemtuzumab 20 and 8 months before encephalitis onset, following 13 years of natalizumab therapy. Three additional patients, all with a thymoma, had serum AChR antibodies without clinical or electrophysiological evidence of myasthenia.

### Clinical Features at the Initial and Subsequent Episodes, and Predictive Factors for Relapse

Clinical features, disease severity, and response to immunotherapy during the initial episode of anti-GABA_A_R encephalitis are summarized in Table 1. Seizures were the main clinical presentation, occurring in 32 of 33 (97%) patients of all ages, of whom 20 of 32 (63%) had status epilepticus. Seizures developed in association with other clinical features in 26 of 33 (79%) patients, mainly cognitive and psycho-behavioral disturbances in adults (17/29, 59% and 9/29, 31%, respectively) and cerebellar symptoms in children (3/4, 75%).

**Figure 1:**
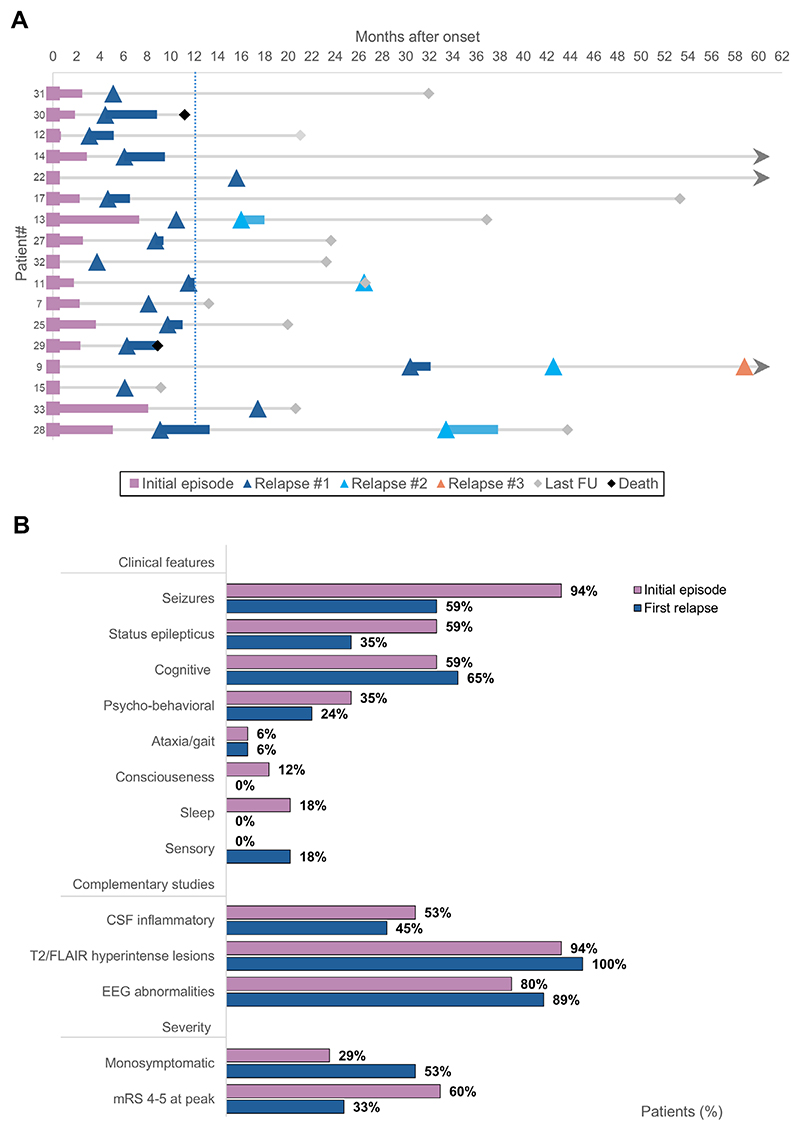
Clinical features of patients with relapses. (A) Swimmer plot showing the disease course of the 17 patients with relapses. Each line on the y-axis represents the disease course of a relapsing patient (numbered as in Table S1). Time spans from clinical onset (time 0) to the last follow-up (gray diamond if alive, black if died) along the x-axis. Three patients (line 4, 5, and 14) had follow-up periods longer than 62 months (gray arrow, duration of follow-up not shown in the graph). The pink squares represent the onset of the initial episode of the disease, whereas triangles show the onset of the relapse episodes (dark blue = first relapse; light blue = second relapse; orange = third relapse). The length of each episode until improvement or stabilization (when available) is represented by a thicker line of the corresponding color. The vertical dashed line marks the 12th month from onset, most relapses occurring within this time period. (B) Comparison between initial episode and first relapse in anti-γ-aminobutyric acid type A receptor (GABA_A_R) encephalitis. Bar chart showing the comparison of clinical symptoms, ancillary test results and severity at the initial episode of the disease and at first relapse in the 17 patients who experienced relapses. None of the comparisons was statistically significant (*p* > 0.05).

Of the 31 patients who survived the initial episode, clinical relapses occurred in 17 (55%), all adults (Fig 1A). The first relapse occurred at a median of 7 months (range, 3–30) after the initial episode. Most relapsed within 1 year (14/17, 82%) and experienced a single relapse (13/17, 76%), although 3 patients had 2 relapses, and 1 had 3 (see Fig 1A). By the time of relapsing symptoms, most patients (12/16, 75%) were not receiving immunotherapy (4 had never received prior immunotherapy given that the initial episode was not recognized as autoimmune encephalitis). Among the 4 on immunotherapy, 3 were tapering steroids, and 1 had received rituximab 6 months prior, with no data on B-cell repopulation.

No significant differences were observed in clinical presentation or ancillary tests between the initial episode and relapse (see Fig 1B). Relapses, however, tended to be more often monosymptomatic (9/17, 53% vs 5/17, 29%; *p* = 0.29) and less severe (mRS, 4–5 in 5/15; 33% vs 9/15; 60% *p* = 0.29) than the initial episode.

Compared to non-relapsing patients, those who relapsed were older (66 vs 55 years, *p* = 0.02), less likely to exhibit movement disorders (0 vs 4/14, 29%; *p* = 0.03), and less likely to have received second-line immunotherapy (2/17, 12% vs 8/14, 57%; *p* = 0.02) (Table S2). At multivariate analysis, older age (odds ratio [OR], 1.063; confidence interval [CI], 1.001–1.128; *p* = 0.047) and lack of treatment with second-line immunotherapy (OR, 9.793; CI, 1.352–70.921; *p* = 0.024) were independent predictors of relapse. After excluding children (all with a non-relapsing course), lack of second-line immunotherapy remained the only independent predictor of relapse (OR, 13.125; CI, 1.924–89.515; *p* = 0.009).

### MRI Features and Changes over the Disease Course

**Figure 2:**
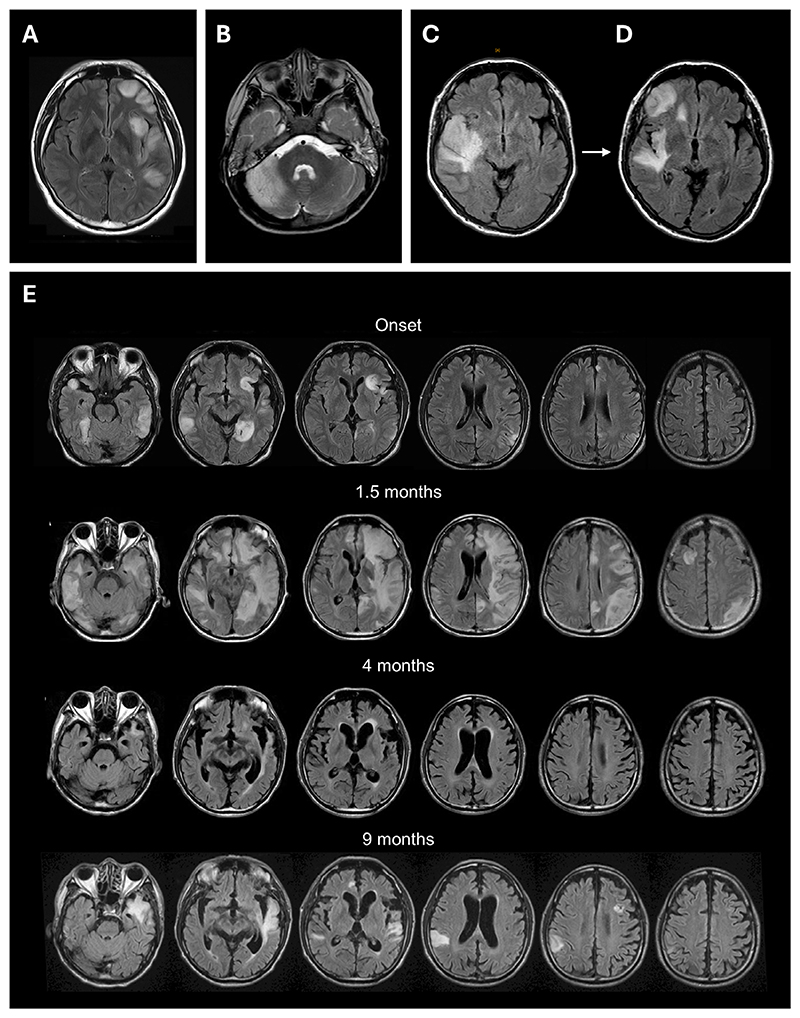
Brain magnetic resonance imaging (MRI) features and changes over time in patients with anti-γ-aminobutyric acid type A receptor (GABA_A_R) encephalitis. (A–D) Initial brain MRIs findings showing: (A) multifocal supratentorial cortico-subcortical T2/fluid-attenuated inversion recovery (FLAIR) hyperintense lesions in the left temporo-fronto-parietal lobes (patient 10); (B) upper right cerebellar hemisphere lesions with minor involvement of left cerebellar folia (patient 1), without contrast enhancement or diffusion restriction (not shown); (C) initial single cortico-subcortical T2/FLAIR hyperintense lesion in the right temporal lobe that evolved at relapse (4 months later) into multiple right frontal lesions with re-expansion of the prior temporal lesion (D) (patient 17). (E) Dynamic changes of brain lesions overtime during the disease course (patient 23). At disease onset, MRI showed multifocal and bilateral T2/FLAIR hyperintense lesions, which worsened during the initial episode eventually merging into confluent areas after 1.5 months. Four months after onset, most of the previous T2/FLAIR abnormalities resolved, and a diffuse brain atrophy developed, predominantly affecting bilateral fronto-temporal lobes. Nine months from onset, the previous inflammatory lesion in the left temporal cortex enlarged, along with the appearance of new small lesions in the parietal and frontal cortices, without apparent worsening of neurological symptoms.

Brain MRI at initial episode was performed a median of 11 days (range, 0–667) after onset and was abnormal in 31 of 33 (94%) patients: 23 (74%, 22 adults, 1 child) had multifocal T2/FLAIR hyperintense cerebral lesions, (16%, all adults) had a single cerebral lesion, and 3 (10%, all children) showed isolated cerebellar inflammatory lesions (Table 1 and Fig 2A–C).

Follow-up MRI images were available for 30 of 33 (91%) patients (2 children and 28 adults). One of the 2 children with cerebellar lesions at onset (see Fig 2B) had residual isolated cerebellar T2/FLAIR hyperintensities at 18 months, with associated slight cerebellar atrophy, whereas the other showed normal findings at 6-months.

In all 28 adults, follow-up MRI demonstrated dynamic lesion changes, including size variation (enlargement or shrinkage) and shifting location (new or resolving lesions), exemplified by patient 20 (see Fig 2E). All 28 developed multifocal supratentorial cortical and subcortical lesions: 22 had this pattern at onset, 5 initially had a single supratentorial lesion, but evolved to multifocal either during the initial episode (in 3) or at relapse (in 2) (see Fig 2C,D), and 1 with a normal initial MRI developed multifocal lesions 6 weeks later, during the same episode. The remaining patient with a normal initial MRI had no follow-up imaging.

In 6 of 28 (21%) patients, new or enlarging cerebral lesions appeared on brain MRI (performed as part of routine clinico-radiological assessment) after a median time of 9.5 months (range, 2–10 months) from the last clinic onset, without new symptoms (“clinically silent lesions”) (see Fig 2E, bottom row). In 2 of 6 patients who were not retreated, these changes preceded neurological relapse by 10 and 30 days, whereas the other 4 of 6 who received immunotherapy based on new MRI findings, remained without new or worsening symptoms.

Among 25 adults with available data, 12 (48%) developed brain atrophy compared to previous scans, sometimes as early as 4 months after onset (see Fig 2E), ranging from focal to diffuse involvement. Demographics, clinical features, tumor presence, and follow-up duration did not differ between those with or without atrophy (*p* > 0.05).

### Other Complementary Studies

Information about other complementary metabolic brain imaging, magnetic resonance spectroscopy (MRS) or pathological studies is shown in Table S1. Brain single photon emission computed tomography (SPECT) or positron emission tomography (PET) demonstrated hyperme-tabolism of T2/FLAIR hyperintense lesions in all the 7 evaluated patients (Fig S1A), with additional adjacent hypometabolic areas in 3, likely related to surrounding edema or network-level functional suppression adjacent to inflamed regions. MRS, performed in 4 patients, revealed increased lactate peak in 1 case^21^ and decreased N-acetylaspartate, inverted choline/N-acetylaspartate ratio, and moderate increase of lactate peaks in the other 2 (Fig S1B), indicating neuronal dysfunction and anaerobic metabolism, a pattern more commonly observed in active inflammatory lesions. In 1 patient, no lactate peak was detected.

Six patients underwent histological studies of the lesions, all showing predominant gliosis. In 1 case, gliosis was accompanied by perivascular CD3^+^/CD8^+^ T-cell infiltration and a few CD20^+^ B-cells (Fig S1C). In another, it was associated with microglial activation, mild perivascular, and parenchymal T-cell infiltration, and later B-cell infiltration at relapse.^22^

Overall, although some of these metabolic abnormalities suggested by the MRS can also be observed during ictal or post-ictal states, the combination of persistent NAA reduction, moderate lactate accumulation, and the histopathological findings of gliosis with mild or focal inflammatory infiltrates makes an inflammatory etiology more plausible in the context of this cohort.

### Long-Term Outcomes (Follow-Up ≥12 Months)

**Figure 3:**
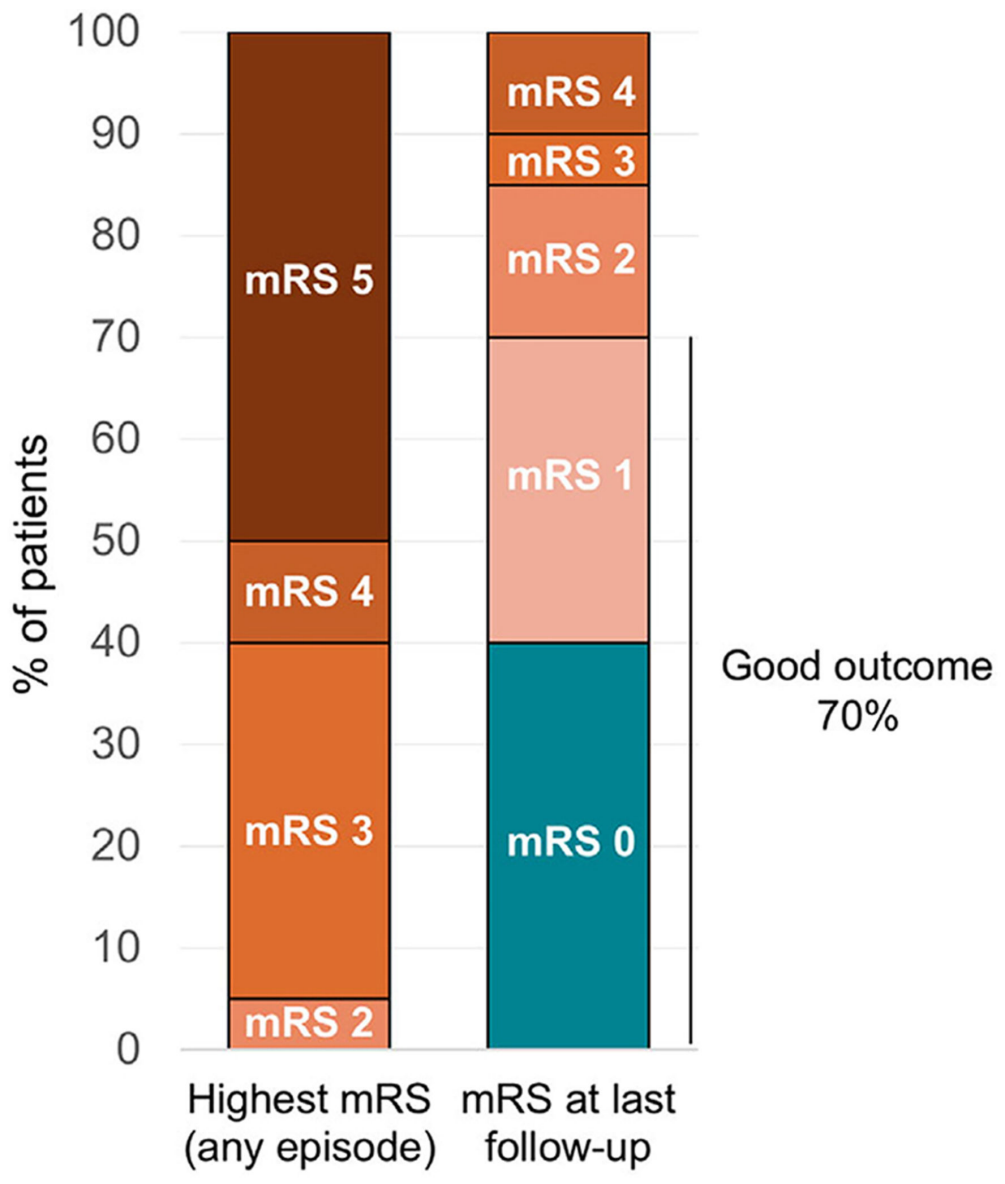
Long-term outcomes in patients with anti-γ-aminobutyric acid type A receptor (GABA_A_R) encephalitis. Grouped bar chart showing modified Rankin scale (mRS) scores at disease peak (highest mRS at any episode) and at last evaluation in patients with long-term follow-up (n = 20).

Overall, 4 of 33 (12%) patients died of causes related to encephalitis: 3 from complications of status epilepticus (2 at the initial episode, 1 at relapse), 1 from sepsis 2 months after relapse. Of the 29 surviving patients, information about long-term outcomes (median follow-up, 32.5 months; range, 12–131) was available for 20 (18 adults and 2 children). Twelve patients (12/20, 60%) had persistent deficits: 9 (9/20, 45%) had cognitive disturbances, either isolated (5 adults and 1 child) or with psycho-behavioral alterations, mild parkinsonism, or motor deficits, 1 adult (5%) had focal seizures and hemispatial neglect, 1 sensory disturbance, and 1 sphincteric dysfunction. Ten (10/14, 71%) patients returned to their pre-morbid occupation (work or school). Outcomes according to mRS scores at last follow-up are shown in Figure 3. Six (6/20, 30%) patients had poor outcome (mRS, 2–5), which was associated with the occurrence of relapse (6/6, 100% vs 6/14, 43% in those with good outcome; *p* = 0.04). Clinical features and results of paraclinical tests were otherwise comparable between the 2 outcome groups (Table 2). However, notably, patients with persistent long-term cognitive deficits tended to be less frequently treated with second-line immunotherapy at the initial episode compared to cognitively unimpaired patients (1/9, 11% vs 6/11, 55%; *p* = 0.07).

### LMO5 Antibody Studies

LMO5 antibodies were assessed in 47 patients with anti-GABA_A_R encephalitis (32 from the present study and 15 from our previous series^1^), and 22 disease controls (5 multiple sclerosis, 17 with suspected neurological disorder) (Fig S2). No LMO5 antibodies were detected in any of the 47 anti-GABA_A_R encephalitis patients (total of 74 samples: 39 sera and 35 CSF, paired in 27 cases), regardless of tumor status, or in the 12 sera and 10 CSF from controls.

## Discussion

This study provides new insights into the clinical, radiological features, and prognosis of anti-GABA_A_R encephalitis. Children present with prominent seizures that can be accompanied by clinical and radiological cerebellar involvement, whereas in adults seizures are typically associated with evolving multifocal supratentorial lesions, which may appear without corresponding clinical changes (“clinically silent lesions”). We also identify novel tumor associations, particularly with gastrointestinal and genitourinary tract malignancies, and report a higher-than-previously recognized relapse rate, affecting over half of patients within the first year, mainly if not treated with second-line immunotherapy. Notably, 60% of patients remain with long-term neurological sequelae, mainly cognitive disturbances, which were associated with relapse and lack of second-line immunotherapy. Finally, LMO5 antibodies appear of limited utility as tumor markers, as none were detected in our cohort.

Seizures are the main clinical manifestations in both children and adults and rarely remain an isolated manifestation. In children, they can frequently associate with cerebellar symptoms and MRI lesions, a radiological pattern unreported in adults in this or prior studies.^1,2,4,5^ In contrast, in adults, comprising 88% of our cohort, seizures are often accompanied by cognitive and/or psycho-behavioral disturbances and multifocal supratentorial MRI lesions. These age-related differences may reflect greater susceptibility of the cerebellum to inflammatory damage during development,^23,24^ consistent with more frequent cerebellar involvement in younger patients in other autoimmune encephalitides, such as anti-alpha-amino-3-hydroxy-5-methyl-4-isoxazole-propionic acid receptor (AMPAR)^25^ and anti-N-methyl-D-aspartate receptor (NMDAR).^19^

Another notable age-dependent difference is that associated tumors occurred only in adults, consistent with the low prevalence of paraneoplastic etiology in pediatric autoimmune encephalitides.^1,19,25,26^ Overall, 60% of adults had an underlying malignancy. Although thymoma remained the most frequent tumor, as previously reported,^1^ a novel finding is that the second most common cancers involved gastrointestinal or genitourinary tract. Whether this association is specific to anti-GABA_A_R encephalitis remains unclear, because tumors in these locations are more common in this age group and may be coincidental.

Multifocal supratentorial cortico-subcortical lesions are a hallmark of anti-GABA_A_R encephalitis. Longitudinal MRI analyses revealed 2 key findings: first, lesions evolved dynamically over time in terms of number, size, and location, likely reflecting shifting underlying inflammation; second, although the initial MRI may show a single lesion, or rarely appear normal, all adult patients eventually developed the characteristic multifocal pattern, either during the initial episode or at relapse. In patients with a single brain MRI lesion, MRS can help differentiate inflammatory from neoplastic processes, whereas metabolic imaging generally adds little diagnostic value. These findings highlight the importance of repeated MRI in suspected anti-GABA_A_R encephalitis, even if early scans are normal or show a solitary lesion. Because biopsies usually reveal nonspecific gliosis, careful radiological follow-up, with MRS when appropriate, may reduce unnecessary invasive procedures.

Clinical relapses occurred in 55% of patients, all adults, mostly within the first year, higher than reported for other autoimmune encephalitides,^19,27,28^ suggesting distinct immune mechanisms. Some patients developed new “clinically silent” MRI lesions, occasionally appearing up to 1 month before symptom recurrence, suggesting ongoing inflammation despite apparent clinical stability, similar to early post-acute anti-leucine-rich glioma inactivated 1 (LGI1) encephalitis.^29^ In our cohort, relapses generally mirrored initial symptoms, although less severe, and occurred mainly in patients off immunotherapy. These findings highlight the need for close MRI monitoring, even in clinically stable patients, as detecting new or evolving lesions can guide maintenance or intensified immunotherapy. This is especially important given the early cerebral atrophy noted in some patients, likely reflecting irreversible neuronal loss.

In our study, over half of patients, mostly adults, had persistent neurological deficits after a median followup of nearly 3 years. Most involved mild to moderate cognitive impairment, whereas only 1 patient had ongoing seizures, consistent with other autoimmune encephalitides such as anti-NMDAR and anti-LGI1, where chronic epilepsy is rare and cognitive sequelae predominate.^27,29–35^

Lack of second-line immunotherapy during the initial episode was associated with higher risk of relapse, which correlated with worse functional recovery (mRS, 2–5). Additionally, persistent cognitive deficits were more frequent in patients untreated with second-line immunotherapy. These findings emphasize the value of early, effective immunotherapy to prevent long-term sequelae, consistent with anti-NMDAR and anti-LGI1 encephalitis, where second-line immunotherapy reduces relapse rate and improves outcomes.^19,36–39^

We did not detect LMO5 antibodies in serum or CSF from any patients with anti-GABA_A_R encephalitis, regardless of tumor status. Two previous studies reported these antibodies in 4 patients with paraneoplastic anti-GABA_A_R encephalitis, suggesting LMO5 release from tumoral cells may trigger cross-reactive autoimmunity.^12,13^ Our findings do not support this and argue against LMO5 antibodies as tumor biomarkers of paraneoplastic anti-GABA_A_R encephalitis.

This study is limited by its retrospective design, relatively small sample size (particularly for pediatric patients and long-term outcomes), and the use of routine MRI scans acquired at non-standardized intervals. In addition, formal neuropsychological tests were not performed in many patients, and the level of recovery was assessed with the mRS scale, which is suboptimal to assess conditions with cognitive sequelae.

Despite these limitations, our findings highlight distinct age-related patterns: encephalitis with cerebellar lesions in children versus encephalitis with evolving multifocal cerebral lesions in adults. Given the high relapse rate and possible subclinical activity, ongoing clinical and MRI surveillance is recommended, even after apparent recovery, alongside early, appropriately intensive immunotherapy. Future studies using CSF cytokine profiling, metabolic and advanced imaging should explore persistent neuroinflammation and its relation to disease activity and outcomes.

## Figures and Tables

**Table 1:** Summary Data on the Patients’ Clinical and Paraclinical Features at the Initial Episode

Clinical features, n (%)	
Prodromes	11/33 (33)
Seizures	32/33 (97)
Status epilepticus	20/32 (63)
Cognitive disturbances	20/33 (61)
Psycho-behavioral changes	10/33 (30)
Sensory symptoms	2/33 (6)
Ataxia/gait/cerebellar disturbances	4/33 (12)
Movement disorders	4/33 (12)
Decreased level of consciousness	5/33 (15)
Sleep disturbances	5/33 (15)
Dysautonomia	4/33 (12)
Isolated symptoms	7/33 (21)
Initial brain MRI, n (%)	
T2/FLAIR hyperintense lesions	31/33 (94)
Cerebral^a^	28/31 (90)
Temporal lobe	24/31 (77)
Frontal lobe	22/31 (71)
Parietal lobe	11/31 (35)
Occipital lobe	8/31 (26)
Hippocampus	5/30 (17)
Diffusion restriction	6/20 (30)
Gadolinium enhancement	6/21 (29)
Cerebellar	3/31 (10)
Bilateral	1/3 (33)
Diffusion restriction	1/3 (33)
Gadolinium enhancement	2/3 (67)
Complementary studies, n (%)	
EEG abnormalities	26/29 (90)
Epileptiform // Slowing	23/26 (88)// 12/26 (46)
CSF abnormalities	20/31 (65)
Pleocytosis (white blood cells>5/ mm^3^)	8/18 (44)
Cell count, median/mm3 (range)	12,5 (7-61)
Hyperproteinorrachia (>45 mg/dL)	12/17 (71)
CSF-specific IgG oligoclonal bands//Increased IgG index	3/15 (20)//3/14 (21)
Treatment, n (%)	
Immunotherapy	28/33 (85)
First-line immunotherapy	28/33 (85)
IVMP	26/28 (93)
Oral steroids	12/28 (43)
PLEX	7/28 (25)
IVIg	11/28 (39)
Response to first-line immunotherapy	
Full	4/26 (15)
Partial	19/26 (73)
None	3/26 (12)
Second-line immunotherapy	10/33 (30)
RTX	6/10 (60)
Azathioprine	4/10 (40)
Others (cyclophosphamide, tacrolimus, cyclosporine, tocilizumab)	4/10 (40)
Response to second-line immunotherapy	
Full	4/7 (57)
Partial	3/7 (43)
Median time to immunotherapy, days (range)	21 (2-246)
Anti-seizure medication	30/30 (100)
Multiple anti-seizure drugs	22/30 (73)
Severity, n (%)	
ICU admission	9/30 (30)
Higher mRS score, median (range)	5 (2–6)

Abbreviations: ICU = Intensive Care Unit; IVIg = intravenous immunoglobulin; IVMP = intravenous methylprednisolone; mRS = Modified Rankin Scale; PLEX = plasma exchange; RTX = rituximab.

a If lesions affected several cerebral lobes, each of the involved lobes was counted separately.

**Table 2:** Comparison of the Clinical and Paraclinical Features Between Patients With Good Versus Poor Outcome at Last Follow-Up

	Good outcome	Poor outcome	*P*
Patients (total 20), n (%)	14 (70)	6 (30)	
Demographics			
Median age at onset, years (range)	51 (4–72)	66 (38–78)	0.09
Females, n (%)	5 (36)	4(67)	0.34
Children, n (%)	2(14)	0	1.00
Comorbidities, n (%)			
Autoimmune diseases	3 (23, n = 13)	4 (67)	0.13
Tumor	9 (64)	2 (33)	0.34
First clinical episode			
Clinical features, n (%)			
Prodromal symptoms	4 (29)	1 (17)	1.00
Seizures	14 (100)	5 (83)	0.30
Status epilepticus	9 (64)	2 (33)	0.34
Cognitive disturbances	9 (64)	2 (33)	0.34
Psycho-behavioral	5 (36)	1 (17)	0.61
Movement disorders	1 (7)	0	1.00
Sleep disorder	3(21)	1 (17)	1.00
Sensory disturbance	2(14)	0	1.00
Dysautonomia	1 (7)	0	1.00
Decreased level of consciousness	1 (7)	2 (33)	0.20
Ataxia/gate/cerebellar disturbances	2(14)	1 (17)	1.00
Initial brain MRI, n (%)			
T2/FLAIR hyperintense lesions	14 (100)	5 (83)	0.30
Cerebral hemisphere(s)	12 (86)	5 (83)	1.00
Cerebellum	2(17, n = 10)	0	0.53
Complementary studies, n (%)			
EEG abnormalities (epileptiform and/or slowing)	10 (91, n = 11)	3 (60, n = 5)	0.21
CSF abnormalities	7 (54, n = 13)	1 (20, n = 5)	0.31
CSF-specific IgG oligoclonal bands	2 (18, n = 11)	0 (n = 4)	1.00
Treatment			
Immunotherapy, n (%)	13 (93)	4(67)	0.20
First-line only, n (%)	7(50)	3 (50)	1.00
First- and second-line, n (%)	6 (43)	1 (17)	0.35
Median time to treatment, days (range)	22.5 (9–246)	40 (3–506)	0.64
Anti-seizure medications	13 (100, n = 13)	5 (83, n = 6)	0.32
Clinical severity			
ICU admission, n (%)	3 (23, n = 13)	2 (33)	1.00
Highest mRS score, median (range)	3 (2-5)	5 (2-5, n = 5)	0.89
Relapses			
Relapse, n (%)	6 (43)	6 (100)	0.04
Clinical course and last follow-up			
Median time from onset to last FU, months	34.5 (12–87)	27 (13–131)	0.78
Cerebral atrophy at last FU MRI, n (%)	3 (25, n = 12)	4 (67)	0.14

Abbreviations: CSF = cerebrospinal fluid; EEG = electroencephalogram; FU = follow-up; ICU = Intensive Care Unit; MRI = magnetic resonance imaging; mRS = modified rankin scale.

## Data Availability

The data that support the findings of this study are available from the corresponding author on reasonable request.
