# Supplementary material for: Relapses, Comorbidities, and Predictors of Outcome in Anti‐GABAA Receptor Encephalitis
Source: Ann Neurol. 2026 Apr 12;100(1):139–50. doi: 10.1002/ana.78208 (PMC7619140; doi:10.1002/ana.78208)
Supplement: Supplementary file 1 — Data S1. Supporting Information. [file ANA-100-139-s001.docx]

**SUPPLEMENTARY MATERIAL**

- Cell-based and western blot assay for LMO5 antibodies.
- Supplementary Table 1: Demographics, clinical and MRI features, immunotherapy, clinical course and outcome of patients with anti-GABA_A_R encephalitis.
- Supplementary Table 2: Comparison of clinical and paraclinical features between patients with and without relapses.
- Supplementary Figure 1: Complementary studies in patients with anti-GABA_A_R encephalitis.
- Supplementary Figure 2: LMO5 antibody testing.
- Supplementary references
- Anti-GABA_A_R encephalitis study group

**Cell-based and western blot assay for LMO5 antibodies**

For cell-based assay, HEK293 cells were transfected with a human Myc–DDK–tagged LMO5/CSRP2 plasmid (catalog number RC201565, accession number NM_001321, OriGene). Twenty-four hours after transfection, cells were fixed with 4% paraformaldehyde for 10 minutes, permeabilized with 0.3% Triton X-100 for 5 minutes and blocked with 1% Bovin Serum Albumin for 2 hours, then incubated overnight with serum (diluted 1:40) or CSF (diluted 1:2) at 4°C. Coverslips were then incubated with Alexa Fluor 488 goat anti-human IgG (diluted 1:1000, 109-545-088, Jackson ImmunoResearch), followed by incubation with a rabbit monoclonal antibody against LMO5/CSRP2 (diluted 1:1000, 1 hour at room temperature, #28601, Cell Signaling Technology) and a secondary antibody (Alexa Fluor 594 goat anti-rabbit IgG, diluted 1:1000, A11012, Invitrogen) for 1h at room temperature. As a transfection control, co-staining with the above mentioned LMO5/CSRP2 antibody and anti-Myc-tag antibody (diluted 1:2000, incubation overnight at 4°C, #2276S, Cell Signaling Technology, followed by a secondary Alexa Fluor 488 goat anti-mouse IgG diluted 1:1000, A11029, Invitrogen) was also performed. Results were imaged under an ApoTome.2 fluorescent microscope (Zeiss, Germany).

For the western blot, the recombinant LMO5/CSRP2 protein (#NBP1-98997, Novus Biologicals) was separated by sodium dodecyl-sulfate polyacrylamide gel electrophoresis (SDS-PAGE) on a 4–12% Bis-Tris gradient gel (50 ng per lane) and transferred to nitrocellulose membranes. After blocking with Skim Milk Powder for 1 hour at room temperature, the membrane were cut in strips and incubated with patient serum (diluted 1:100) or LMO5/CSRP2 antibody (diluted 1:1000, #28601, Cell Signaling Technology) overnight at 4°C. Strips were then washed and incubated with horseradish peroxidase-conjugated secondary antibodies (anti-human IgG, diluted 1:1000, #109-035-088, Jackson ImmunoResearch or anti-rabbit IgG, diluted 1:1000, #NA934, Cytiva) for 1 hour at room temperature. The reactivity was developed with ECL chemiluminiscence (ECL™ Western Blotting Detection Reagents, Cytiva, RPN2209).

LMO5 antibodies were determined in serum and CSF samples from patients with anti-GABA_A_R encephalitis (both identified from this study and from our previous cohort, all fulfilling the inclusion criteria) ^1^ and disease controls, including multiple sclerosis and suspected autoimmune neurological disorders with extensive negative neural antibody testing (using immunohistochemistry on rat brain/cerebellum, immunodot and CBAs).

**eTable 1.** **Demographics, clinical and MRI features, immunotherapy, clinical course and outcome of patients with anti-GABA_A_R encephalitis.**

| **Pt** | **Age/**  **Gender** | **Tumor association and time of diagnosis with respect to encephalitis**  **Autoimmune comorbidities** | **Prodromal symptoms** | **Clinical features at initial episode; mRS at peak;**  **GABA_A_R antibody status in serum/CSF at initial episode** | **T2/FLAIR hyperintense lesions at first brain MRI; diffusion restriction; Gd+ contrast enhancement**  **Complementary tests** | **Immunotherapy** | **Relapse: main clinical features; mRS at peak**  **GABA_A_R antibody status in serum/CSF at relapse** | **Last FU (time from onset, months); mRS** |
| --- | --- | --- | --- | --- | --- | --- | --- | --- |
| 1 | 4/M | No tumor | None | Insomnia followed by progressive speech disturbances leading to mutism, seizures (focal) and ataxia  S: +, CSF: + | Upper region of the right cerebellar hemisphere, with tumefactive effect, and less marked alterations in some left cerebellar folia; no diffusion restriction; no CE | IVMP, IVIg (acute phase and monthly administration) | None | 22; mRS 1 |
| 2 | 5/M | No tumor | Flu-like symptoms | Onset with ataxia and vertigo, followed by refractory seizures (focal and tonic-clonic) evolving into SE and dysautonomia (bradycardia, hypotension); resuscitated from a first cardiorespiratory arrest, patient died during SE complicated with sepsis; mRS 6  S: +; CSF: N/A | Right cerebellar hemisphere; diffusion restriction; slight leptomeningeal CE | IVMP, IVIg | Not applicable | 1; mRS 6 |
| 3* | 6/M | No tumor | Headache | Fever and refractory seizures (focal) evolving into SE (refractory), followed by involuntary movements in right lip and upper limb, hypertension, cognitive impairment (including speech disturbances), extremity tremors and cerebellar ataxia with left hemibody hyperreflexia; mRS 5  S: +, CSF: + | Posterior region of the left cerebellar hemisphere, mild tumefactive effect; no diffusion restriction; some CE foci | IVMP, oral steroids, PLEX, cyclosporine, azathioprine and monthly IVIg | None, but the patient had seizures after the acute episode during steroid tapering | 70; mRS 0 |
| 4 | 12/M | No tumor | Headache, fever, and vomiting | Seizures, cognitive, psycho-behavioral (incoherent speech, hallucinations), movement disorders (dystonia and oral movements), sleep disorder, progressing to decreased level of consciousness; mRS 5  S: N/A, CSF: + | Bilateral (L>R) cortico-subcortical lesions in temporal, frontal, and parietal lobes; DWI N/A; frontal and parietal areas of minimal CE | IVMP, IVIg | None | 6; mRS 2 |
| 5 | 23/F | No tumor  N/A | None | Seizures (tonic-clonic; focal); mRS 2  S: +, CSF: + | Multiple lesions with predominant cortical involvement in the bilateral frontal lobes and in the right temporal lobe; one lesion with diffusion restriction; no CE | IVMP, IVIg, RTX, azathioprine | None | 87; mRS 0 |
| 6 | 27/F | No tumor | Low-grade fever and disorientation | Dysautonomia and seizures (tonic-clonic) evolving into SE (convulsive and NCSE), eventually complicating with sepsis and leading to death; mRS 6  S: +, CSF: + | None | None | Not applicable | 9; mRS 6 |
| 7 | 38/F | Multiple sclerosis (treated with Natalizumab for 13 years, then Alemtuzumab 20 and 8 months before encephalitis); hypothyroidism/thyroiditis | None | Seizures (focal at onset, then tonic-clonic) followed by cognitive impairment and evolving into SE (focal motor, NCSE); mRS 5  S: +, CSF: + | Multiple cortico-subcortical in the left fronto-insular and parietal lobes and right temporo-parietal-insular lobes; cortical diffusion restriction; no CE | IVMP, PLEX, IVIg, tocilizumab, RTX | Cognitive and gait ataxia; mRS 3 | 13; mRS 2 |
| 8 | 42/M | Thymoma (surgical treatment 6 months before encephalitis onset) | None | Seizures (tonic-clonic) followed by myoclonic jerks in his left upper limb (focal motor SE); mRS 3  S: +, CSF: + | Multiple cortico-subcortical lesions in the right fronto-temporal lobe; no diffusion restriction; no CE | IVMP, oral steroids, azathioprine | None | 41; mRS 0 |
| 9 | 43/M | Thymoma (diagnosed in concomitance to relapse; surgical treatment)  Anti-AChR antibodies | None | Progressive cognitive impairment (almost 2 years), followed by seizures; mRS 3 | Multiple brain lesions; DWI N/A; CE N/A | IVMP, oral steroids | 1^st^: Psycho-behavioral (fatigue); 2^nd^: Psycho-behavioral (irritability) and seizures; 3^rd^: Psycho-behavioral (irritability); mRS 3.  S: +, CSF: + | 74; mRS 0 |
| 10* | 44/M | Thymoma (diagnosed at encephalitis onset; surgical treatment) | None | Progressive aphasia, followed by sensory impairment in the right face, right side myoclonic jerks (focal motor SE) and cognitive disturbances, then tonic-clonic seizures; mRS 5  S: +, CSF: + | Multiple cortico-subcortical lesions in the left temporo-fronto-parietal lobes and in the right frontal lobe; no diffusion restriction (DWI increased, but ADC increased as well); CE N/A  Brain FDG-PET: hypermetabolism in the left cerebral hemisphere with focal reduction in the left temporal lobe  Magnetic resonance spectroscopy: no lactate peak | IVMP, oral steroids | None | 32; mRS 1 |
| 11 | 49/M | Thymoma (diagnosed at encephalitis onset; no treatment) | Headache | Cognitive (including speech disturbances) and seizures (tonic-clonic); mRS 5  S: +, CSF: + | Multiple cortico-subcortical lesions (predominant involvement of subcortical areas) in the bilateral temporal, frontal and parietal lobes; no diffusion restriction; subtle CE in the right temporal cortex | IVMP, oral steroids | 1^st^: Sensory symptoms; mRS 1. 2^nd^: Seizures/SE, mRS 5. | 11; mRS 5 (during acute phase of 2^nd^ relapse) |
| 12 | 51/F | Thymoma (diagnosed 17 months after encephalitis onset; surgical treatment)  Anti-AChR antibodies | Herpes labialis | Right facial twiching (SE focal motor); mRS 3  S: +; CSF: NEG | Multiple bilateral cortico-subcortical lesions in the temporal lobes; no diffusion restriction; CE N/A | IVMP | Limb dysesthesia: mRS 3 | 20; mRS 0 |
| 13 | 51/F | Thymoma (diagnosed at encephalitis onset; surgical treatment); | None | Onset with psycho-behavioral alterations, followed by insomnia, hyporexia and weight loss, psychosis, cognitive disturbances and refractory seizures; mRS 4  S: +, CSF: + | Multiple cortical-subcortical lesions in both frontal lobes and right temporal lobe; no diffusion restriction; no CE  Brain FDG-PET: hypermetabolism in frontal lobes, hypometabolism in temporal-parietal and fronto-basal regions. | IVMP, PLEX, oral steroids | 1^st^: Seizures (enlargement of frontal lesion); mRS 1. 2^nd^: Seizures/SE, psycho-cognitive, motor weakness; mRS 5  S: +, CSF: + | 36; mRS 1 |
| 14 | 53/M | Thymoma (diagnosed at encephalitis onset; surgical treatment + RT; tumor recurrence after 53 months, no treatment) | None | Vertigo, nausea and nystagmus, followed by seizures; mRS 2  S: +, CSF: + | Multiple cortico-subcortical lesions in temporal lobe, frontal lobe, parietal lobe and hippocampus; no diffusion restriction; no CE | IVMP | Cognitive, psycho-behavioral; mRS 5 | 68; mRS 4 |
| 15 | 54/M | Tubulo-villous adenoma of recto-sigma with low grade dysplasia (diagnosed in concomitance to encephalitis onset; treatment N/A) | None | Movement disorders, headache, decreased hearing and tinnitus (bilateral veiling of the middle ear on brain MRI), seizures, SE, cognitive disturbances (after SE); mRS 5  S: +; CSF: + | Multiple cortico-subcortical lesions in the bilateral frontal, temporal, parietal and left occipital lobes; partial diffusion restriction; no CE.  Brain PET: multifocal hypermetabolic areas | IVMP, IVIg, RTX | None | 10; mRS 0 |
| 16 | 56/M | Metastatic prostate adenocarcinoma (diagnosed before encephalitis onset; treatment N/A)  Chron’s disease | None | Seizures (focal), SE, cognitive (including speech disturbances), psycho-behavioral, anorexia, weight loss, headache; mRS 3  S: NEG; CSF:+ | Multiple cortical and cortico-subcortical lesions in temporal, frontal, occipital lobes and hippocampus; DWI N/A; some areas with CE (one lesion compatible with ischemia; concomitant skull bone metastasis). | IVMP, IVIg | None | 12; mRS 1 |
| 17 | 57/M | Thymoma (diagnosed at encephalitis onset; surgical treatment) | Headache | Persistent headache, followed by insomnia, cognitive dysfunction and psycho-behavioral alteration (NCSE); mRS 4  S: +, CSF: + | Single cortico-subcortical lesion in the right temporal lobe; no diffusion restriction; inhomogeneous cortical CE  IMP-SPECT: focal hyperperfusion in right temporal lobe | IVMP, IVIg | Cognitive, left-hand dysesthesia, NCSE; mRS 3 | 52; mRS 1 |
| 18 | 60/F | No tumor | None | Headache and episodes of lateropulsion, sensory and cognitive disturbances, likely seizure manifestations (EEG with epileptiform discharge); mRS 3  S: N/A; CSF: + | Multiple cortico-subcortical lesions in bilateral cerebral hemispheres; no diffusion restriction; no CE | IVIg, azathioprine | None | 33; mRS 0 |
| 19 | 60/M | Thymoma (diagnosed in concomitance to encephalitis onset; surgical treatment but worsening of pulmonary lesions after 4 months, probable thymoma metastasis)  Anti-AChR-ab positive MG | None | Fever, episodes of psycho-behavioral alterations and cognitive impairment, likely seizure manifestations (EEG with epileptiform discharges); mRS 3  S: +, CSF: + | Multiple cortico-subcortical lesions in bilateral frontal lobes, right temporal lobe and hippocampus; no diffusion restriction (only slight increase in DWI signal in cortex, ADC N/A); CE N/A | IVMP, PLEX, IVIg, oral steroids, RTX, tacrolimus | None | 6; mRS 0 |
| 20 | 61/M | No tumor  Biermer’s disease; thyroiditis | None | Cognitive (with speech disturbances), jerks on the left side of the face (SE focal motor - EPC), right hand choreoathetosis, intestinal pseudo-obstruction, followed by respiratory failure and deterioration of consciousness; mRS 5  S: +, CSF: + | Multiple cortico-subcortical in the bilateral frontal, temporal, parietal, occipital lobes and cingulate gyrus; no diffusion restriction (increased signal in DWI, ADC isointense); no CE  Magnetic resonance spectroscopy: decreased N-acetyl aspartate, choline/N-acetyl aspartate inversion, lactate peak.  Brain biopsy: non-specific reactive gliosis | IVMP, PLEX, IVIg, oral steroids, RTX | None | 5; mRS 0 |
| 21 | 62/M | No tumor | Nausea, dizziness | Seizures, cognitive; mRS 3  S: +; CSF: + | Multiple cortico-subcortical lesions in the bilateral temporal, frontal, occipital, insula lobes and hippocampus; diffusion restriction; no CE | Oral steroids | None | 3; mRS 1 |
| 22* | 63/M | Ossifying thymoma (diagnosed at relapse: surgical treatment)  AChR-ab positive ocular MG; Neuromyotonia | None | Seizures (one year history of cramps, ptosis and diplopia); mRS N/A  S: +, CSF: + | Multifocal cortico-subcortical lesions in frontal, parietal, temporal lobe; DWI and CE N/A | None | Cognitive (6-month evolution), myoclonus, seizures; mRS 4 | 131; mRS 2 |
| 23 | 65/M | Multiple myeloma (diagnosed 21 months before encephalitis onset, treated with ASCT 1 y before followed by and chemotherapy) | Headache and vomit | Progressive encephalopathy with headache, cognitive impairment, psycho-behavioral changes and speech disturbances (NCSE); mRS 5  S: +, CSF: + | Multiple cortico-subcortical lesions in bilateral temporal and parietal lobes, and right frontal and occipital lobes; mild increased DWI signal along the gyri of the affected cortex (probable shine through effect); CE N/A | IVMP, IVIg, PLEX | None | 10; mRS 2 |
| 24 | 66/F | Thymoma (diagnosed in concomitance to encephalitis onset; surgical treatment) | None | Seizures (focal, vertigo)/SE; mRS 2  S:+; CSF: N/A (samples tested 2 years after onset) | Cortico-subcortical lesion in the right temporal lobe; DWI N/A; no CE  Brain biopsy: reactive gliosis | None | Speech disturbances; mRS 2 | 6; mRS 0 |
| 25 | 66/M | No tumor | None | Seizures (focal) and SE (focal motor - EPC); mRS 3 | Single cortico-subcortical lesion in the right frontal lobe; no diffusion restriction; minimal peripheral CE  Magnetic resonance spectroscopy: decreased N-acetyl aspartate peak and relatively increase lactate peak  Brain lesion histology: gliosis,  infiltration of CD3+/CD8+ T-cells in the vascular space, and few CD8-, CD4- or CD20-positive cells in the parenchyma | None | Seizures/SE; mRS 3  S: +, CSF: NEG | 19; mRS 1 |
| 26* | 67/F | GIST (surgical treatment 1 year before encephalitis onset)  Thyroiditis | None | Dysesthesia in left lower limb (improved after levetiracetam), followed by cognitive disturbances, tinnitus and seizures (tonic-clonic) with a subsequent delirious state (SE - NCSE); mRS 5  S: +, CSF: + | Single lesion of the right temporal operculum involving the insula; no diffusion restriction; no CE  Magnetic resonance spectroscopy: elevated lactate signals and Lac/creatine ratio | IVMP, oral steroids, oral cyclophosphamide | None | 30; mRS 0 |
| 27 | 69/F | No tumor  Bullous pemphigoid | None | Psycho-behavioral alterations (anorexia, anxiety), insomnia, headache and nausea, cognitive dysfunction (including speech disturbances), fever, seizures (focal and tonic-clonic) and SE (NCSE and focal motor); mRS 5  S: +, CSF: + | Normal brain MRI; FU: Multiple cortico-subcortical lesions in the bilateral frontal and temporal lobes and left hippocampus; some lesions with cortical diffusion restriction; no CE | IVMP | Seizures/SE (presenting as psycho-behavioral symptoms); mRS 3 | 23; mRS 2 |
| 28 | 72/M | Prostate adenocarcinoma (diagnosed at relapse; surgical treatment); thymoma (diagnosed at relapse; surgical treatment)  Systemic Lupus Erythematosus | Abdominal pain and vomiting | Psycho-behavioral changes, followed by seizures (focal evolving into tonic-clonic) and NCSE; after SE, evidence of cognitive disturbances. | Multiple cortico-subcortical lesions in bilateral cerebral hemispheres (R>L) involving temporal lobes and frontal lobe; cortical areas of diffusion restriction; no CE.  Brain PET: hypermetabolism corresponding to MRI lesions and adjacent hypometabolic areas | IVMP, PLEX, RTX | 1^st^: Seizures and cognitive; mRS 5. 2^nd^: Seizures/SE and cognitive; mRS 5.  S: +; CSF: + | 43; mRS 0 |
| 29 | 74/M | Thymoma (diagnosed in concomitance to encephalitis onset; surgical treatment)  Anti-AChR antibodies | None | Psycho-behavioral changes and seizures (focal, tonic-clonic) evolving into SE (NCSE); mRS 5  S: + (only CBA); CSF: NEG | Multiple lesions with predominant cortical involvement in the bilateral cerebral hemispheres (mainly right temporo-occipital lobe); no diffusion restriction; no CE | IVMP, oral steroids | Cognitive and seizures/SE, resistant to treatment and leading to death for septic shock; mRS 6  S: +, CSF: + | 8; mRS 6 |
| 30 | 76/F | Esophageal cancer (surgical treatment 2 years before encephalitis onset) | None | Headache, myoclonic jerks (SE focal motor), aphasia and right hemispatial neglect, evolving into akinetic mutism (SE - NCSE); when improved, persistence of cognitive impairment (including aphasia); mRS 5  S: +, CSF: + | Bilateral cortico-subcortical lesions in frontal, temporal, parietal and occipital lobes; mild DWI hyperintensity (probable shine-through effect); no CE  IMP-SPECT: focal hyperperfusion in the right frontal and temporal regions  Brain biopsy: steroid-responsive subacute inflammatory encephalopathy of unknown cause | IVMP | Seizures/SE; mRS 5 | 11; mRS 6 (died for septic shock) |
| 31* | 78/F | No tumor | Dizziness, vomiting, anorexia, low grade fever | Headache and decreased level of consciousness evolving into akinetic mutism; mRS 5  S: +, CSF: + | Bilateral cortico-subcortical lesions in frontal, temporal and parietal lobes; DWI showed hypointense core of lesion with high peripheral signal; no CE  Brain biopsy: gliosis and microglial activation, mild perivascular lymphocytic infiltrate | IVMP | Seizures/SE, cognitive, psycho-behavioral; mRS 5 | 31; mRS 4 |
| 32 | 78/F | No tumor  Thyroiditis | None | Seizures (focal); mRS 2 | Cortico-subcortical lesions in the left temporal and frontal lobes; cortical temporal diffusion restriction; mild cortical temporal CE  11C‑methionine PET: mild accumulation of the tracer corresponding to MRI lesion | None | Seizures, cognitive impairment and left tactile extinction; these symptoms improved but were followed by another episode of seizures/SE and wandering behaviors (< 2 months); mRS 5  S: +, CSF: + | 22; mRS 3 |
| 33 | 82/F | No tumor | None | Nausea/dizziness, seizures (focal), SE, cognitive; mRS 5 | Lesion in the left medial frontal cortex; DWI N/A; CE N/A  Brain biopsy: increased number of small-sized glial cells, with low or middle cell density and micro-clustering in the surroundings of neurons; immunohistochemically, these cells were positive for Olig2, and positive for GFAP in a scattered manner; MGMT silencing appeared weakly positive | IVMP, oral steroids | Cognitive; mRS 3  S: +; CSF: + | 20; mRS 3 |

*Published case reports (Ueno et al., 2023^2^; Fukami et al., 2017; ^3^ Kurihara et al., 2020; ^4^ Valle et al., 2021; ^5^ Hashimoto et al., 2023^6^) but with updated clinical information. Abbreviations: CE: contrast enhancement; CSF: cerebrospinal fluid; EPC: epilepsia partialis continua; F: female; FLAIR: fluid-attenuated inversion recovery; FDG: fluorodeoxyglucose; FU: follow-up; Gd: gadolinium; GFAP: glial fibrillary acidic protein; IMP-SPECT: iodine-123 n-isopropyl-p-iodoamphetamine single photon emission computed tomography; IVIG: intravenous immunoglobulin; IVMP: intravenous methylprednisolone; M: male; MRI: magnetic resonance imaging; mRS: modified Rankin scale; N/A: not available; NCSE: non convulsive status epilepticus; NEG: antibody status negative; PET: positron emission tomography; PLEX: plasma exchange; RTX: Rituximab; SE: status epilepticus.

**eTable 2. Comparison of clinical and paraclinical features between patients with and without relapses.**

|  | **Relapse** | **No relapse** | **p value** |
| --- | --- | --- | --- |
| Patients (tot. 31), n (%) | 17 (55) | 14 (45) |  |
| Follow-up, months (range) | 23 (8-131) | 17 (3-87) | 0.25 |
| **Demographics** | | | |
| Median age at onset, years (range) | **66 (38-82)** | **55 (4-67)** | **0.02** |
| Females, n (%) | 9 (52) | 3 (21) | 0.14 |
| Children, n (%) | 0 | 3 (21) | 0.08 |
| **Comorbidities, n (%)** | | | |
| Autoimmune diseases | 6 (35) | 3 (23, n=13) | 0.69 |
| Tumor | 11 (65) | 7 (50) | 0.50 |
| **First clinical episode** | | | |
| **Clinical features, n (%)** | | | |
| Prodromal symptoms | 5 (29) | 5 (36) | 1.00 |
| Seizures | 16 (94) | 14 (100) | 1.00 |
| Cognitive disturbances | 10 (59) | 10 (71) | 0.70 |
| Psycho-behavioral | 6 (35) | 4 (29) | 1.00 |
| Movement disorders | **0** | **4 (29)** | **0.03** |
| Sleep disorder | 3 (18) | 2 (14) | 1.00 |
| Sensory disturbance | 0 | 2 (14) | 0.20 |
| Dysautonomia | 0 | 2 (14) | 0.20 |
| Decreased level of consciousness | 2 (12) | 3 (21) | 0.64 |
| Gait/balance disturbances | 1 (6) | 2 (14) | 0.58 |
| **Brain MRI, n (%)** | | | |
| T2/FLAIR hyperintense lesions | 17 (100) | 14 (100) | 1.00 |
| Cerebral hemisphere(s) | 17 (100) | 12 (85) | 0.58 |
| Multifocal | 12 (71) | 11 (79) | 0.35 |
| Bilateral | 10 (59, n=16) | 10 (71) | 0.71 |
| Cerebellum | 0 (n=15) | 2 (15, n=13) | 0.20 |
| MRI normalization after first episode | 0 | 2 (14) | 0.19 |
| **Complementary studies, n (%)** | | | |
| EEG abnormalities (epileptiform and/or slowing) | 12 (80, n=15) | 12 (100, n=12) | 0.23 |
| CSF abnormalities | 8 (53, n=15) | 10 (71) | 0.45 |
| CSF-specific IgG oligoclonal bands | 1 (7, n=14) | 1 (11, n=9) | 1.00 |
| **Treatment** | | | |
| Immunotherapy, n (%) | 13 (77) | 14 (100) | 0.11 |
| First-line immunotherapy, n (%) | 13 (77) | 14 (100) | 0.11 |
| First- and second-line immunotherapy, n (%) | **2 (12)** | **8 (57)** | **0.02** |
| Median time to immunotherapy, days (range) | 77 (3-216) | 133,5 (2-246) | 0.57 |
| Anti-seizure medications | 15 (94, n=16) | 13 (100, n=13) | 1.00 |
| **Clinical severity** | | | |
| ICU admission, n (%) | 3 (19, n=16) | 5 (39, n=13) | 0.41 |
| mRS at peak, median (range) | 4.5 (2-5, n=16) | 4 (2-5) | 0.98 |

Abbreviations: CSF: cerebrospinal fluid; ICU: intensive care unit; MRI: magnetic resonance imaging; mRS: modified rankin scale.

**eFigure 1. Complementary studies in patients with anti-GABA_A_R encephalitis.**

**
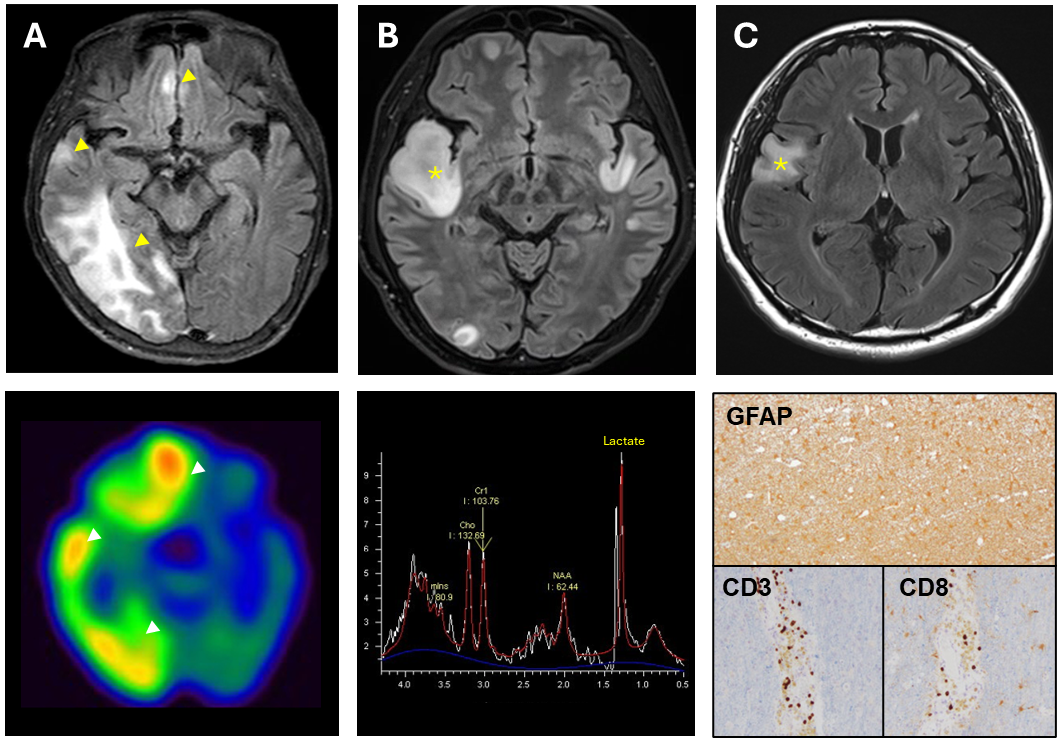
**

(**A**) IMP-single photon emission computed tomography (SPECT) of patient #30 shows hypermetabolic areas (bottom, arrowheads) corresponding to T2/FLAIR hyperintense lesions in the right hemisphere on brain MRI (top, arrowheads).

(**B**) Short echo time magnetic resonance spectroscopy (bottom) of patient #20 shows a decrease in N-acetylaspartate (NAA), choline/NAA inversion, and a lactate peak corresponding to the T2/FLAIR hyperintense lesion in the right temporal lobe (top, asterisk).

(**C**) Brain biopsy (bottom) of the T2/FLAIR hyperintense temporal lesion (top, asterisk) of patient #25 shows gliosis (glial fibrillary acidic protein [GFAP] staining), infiltration of CD3+/CD8+ T-cells in the vascular space (CD3 and CD8 staining), and few CD8-, CD4- or CD20-positive cells in the parenchyma (not shown).

**eFigure 2. LMO5 antibody testing**


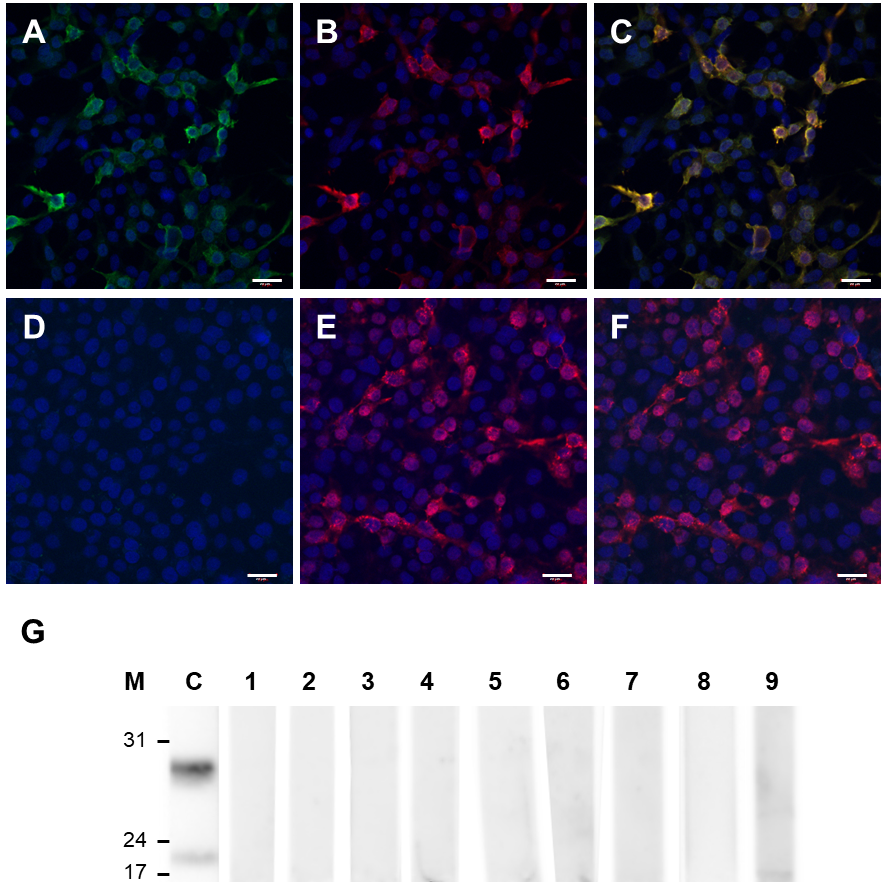


(**A-F**) Cell-based assay with HEK293 cells expressing Myc-tagged LMO5. Cells were immunolabelled with commercial antibodies against Myc-tag (**A**, *green*) and LMO5 (**B** and **E**, *red*) to confirm LMO5 expression and the signal co-localization in transfected cells (**C**, *merged*). No immunoreactivity was observed with a serum from a patient with anti-GABA_A_R encephalitis (**D**) on HEK293 cells expressing LMO5 (**E**, *red* and **F,** merged reactivities). Nuclei (*blue*) were counterstained with 40,6-diamidino-2-phenylindole (DAPI). Scale bar = 20 μm.

(**G**) Western blot analysis of His-tagged LMO5. Lane M: molecular weight marker (kDa); lane C: LMO5 detected by the commercial anti-LMO5 antibody; lanes 1-9: no identical bands were detected with patients’ serum (lanes 1-3: sera from patients from Spatola et al.; ^1^ lanes 4-9: sera from patients #19, #20, #5, #29, #28, #21 from this study).

**Supplementary references**

1. Spatola M, Petit-Pedrol M, Simabukuro MM, et al. Investigations in GABAA receptor antibody-associated encephalitis. Neurology. 2017 Mar 14;88(11):1012-20.

2. Ueno H, Iizuka T, Tagane Y, et al. Focal hyperperfusion and elevated lactate in the cerebral lesions with anti-GABAaR encephalitis: A serial MRI study. J Neuroradiol. 2020 May;47(3):243-6.

3. Fukami Y, Okada H, Yoshida M, Yamaguchi K. [Successful combination immunotherapy of anti-gamma aminobutyric acid (GABA)(A) receptor antibody-positive encephalitis with extensive multifocal brain lesions]. Rinsho Shinkeigaku. 2017 Aug 31;57(8):436-40.

4. Kurihara M, Sasaki T, Sakuishi K, et al. Isolated seizure as initial presentation of GABA(A) receptor antibody-associated encephalitis. J Neurol Sci. 2020 Mar 15;410:116666.

5. Valle DAD, Santos M, Spinosa MJ, Telles BA, Prando C, Cordeiro ML. GABAA receptor encephalitis associated with human parvovirus B19 virus infection: Case report. Medicine (Baltimore). 2021 Jun 11;100(23):e26324.

6. Hashimoto K, Nakamura T, Fujita Y, et al. Coupling of Cortical Hyperintense Signals and Increased Glucose Metabolism in a Case of Anti-GABA(A) Receptor Antibody-associated Encephalitis. Intern Med. 2023 Dec 1;62(23):3545-8.

**ANTI-GABA_A_R ENCEPHALITIS STUDY GROUP**

| **Surname, name** | **E-mail** | **Affilitation** |
| --- | --- | --- |
| Araki, Amane | amane@nagoya2.jrc.or.jp | Department of Neurology, Japanese Red Cross Aichi Medical Center Nagoya Daini Hospital, Nagoya, Japan |
| Hashimoto, Kentaro | g.hashimotokentarou@gmail.com | Geriatrics Research Institute and Hospital, Maehashi, Japan |
| Itagaki, Yuya | yta@fmu.ac.jp | Fukushima Medical University Hospital, Fukushima, Japan |
| Marini, Sofia | sofiamarini97@gmail.com | Department of Neuroscience, Catholic University of the Sacred Heart, Rome, Italy |
| Morimoto, Yuko | yk.konno@gmail.com | Department of Neurology, Chikamori Hospital, Kochi, Japan |
| Morishita, Naoki | naoki.morishita@hospital.yaizu.shizuoka.jp | Department of Neurology, Yaizu City Hospital, Yaizu, Japan |
| Nishi, Ryoji | nishi-r@gc4.so-net.ne.jp | Department of Neurology, Japan Community Health Care Organization Chukyo Hospital, Nagoya, Japan |
| Soejima, Naoko | nsoejima98@gmail.com | Department of Neurology, Kyushu Rosai Hospital, Kitakyushu, Japan |
| Takenouchi, Akiyuki | takenouchi@hama-med.ac.jp | Department of Neurology, Hamamatsu University Hospital, Hamamatsu, Japan |
| Ueno, Hiroki | uhiroki@mac.com | Hiroshima City Hiroshima Citizens Hospital, Hiroshima, Japan |
